# Dynamics of Leptospirosis Transmission Within Urban Norway Rat (*Rattus norvegicus*) Populations in Densely Populated French Areas: Implications for Public Health

**DOI:** 10.1155/tbed/3451406

**Published:** 2025-02-22

**Authors:** Sionfoungo Soro, Karine Groud, Ambre Fafournoux, Elodie Monchatre-Leroy, Angeli Kodjo, Sébastien Lefebvre, Nolan Chatron, Virginie Lattard

**Affiliations:** ^1^USC1233 RS2GP, INRAe, VetAgro Sup, University of Lyon, Marcy l'Étoile, France; ^2^Laboratoire De La Rage Et De La Faune Sauvage De Nancy (LRFSN), Anses, Malzéville, France

## Abstract

Leptospirosis, a bacterial zoonosis with a worldwide distribution, represents a major public health challenge. It is caused by the spirochete Leptospira, whose main reservoir in urban environments is the brown rat (*Rattus norvegicus*). Understanding the transmission dynamics of this disease within a rat population is essential for controlling the risk of human infection. In this study, an original capture method was used to analyze variations in carriage and bacterial load according to age in two distinct populations of brown rats, to provide a better understanding of the transmission routes of Leptospira within a population. A total of 508 rats were captured from all age categories, from newborns to very young rats (representing 18% of the animals) to very old rats (representing 21% of the animals). The overall prevalence of leptospirosis was between 30% and 50%, depending on the population. A single strain was identified in both studied populations: *Leptospira interrogans* belonging to the Icterohaemorrhagiae serogroup and the Icterohaemorrhagiae serovar. Surprisingly, in both populations, our study reveals a sudden change in the prevalence at 300/400g, jumping from 20 to 30% to over 75%. Moreover, none of the 98 fetuses collected from 13 pregnant females infected with Leptospira was detected as infected. This sudden change and the absence of infected fetus demonstrate the major role of horizontal transmission in the dynamics of leptospirosis infections and minimize the importance of vertical transmission.

## 1. Introduction

Leptospirosis, a zoonosis of worldwide occurrence [1], caused by a bacteria spirochete of the *Leptospira* genus, is a real public health issue, with significant consequences for both humans and animals [2, 3]. In humans, the symptoms are highly variable, which make diagnosis complex. They range from a mild flu-like syndrome to severe forms with multiorgan failure, which can result in pulmonary hemorrhage, jaundice, and meningitis, often leading ultimately to respiratory and/or renal failure with high mortality rates [4–6]. It is estimated that approximately one million individuals worldwide are afflicted by this disease, which causes nearly 60,000 deaths annually [7]. The variability in clinical signs, which is a feature of many other disorders, and the lack of awareness among clinicians contribute to misdiagnosis of the disease, despite the existence of commonly used diagnostic tests [1, 8]. In animals, the disease can result in significant economic losses for farmers due to abortions and stillbirths [3].

Leptospirosis, previously regarded as a bacterial disease predominantly affecting rural communities [9], is now recognized as a re-emerging public health concern in numerous countries. The disease is becoming increasingly prevalent in developed countries where certain leisure activities [10] and the presence of unhealthy urban environments [11] contribute to its spread, posing a growing threat to urban health. It is notable that metropolitan France is not exempt from this trend, with one of the highest incidence rates in Europe [9]. In 2014 ad 2015, the estimated incidence rate was 1 case per 100,000 inhabitants [12].

Transmission to humans or animals occurs via direct or indirect contact with infected animals' urine or body secretions or via contaminated environments [2, 8, 13]. A variety of animal species, including fish, amphibians, reptiles, birds, and mammals, have been identified as hosts for *Leptospira*, as evidenced by numerous studies [2, 8, 14]. Some species are susceptible and considered accidental hosts, which present a severe form of the infection with severe clinical signs but excrete a limited quantity of *Leptospira*. Others are chronic, asymptomatic carriers, which host the bacteria in the kidney and excrete it abundantly and intermittently in their urine. These chronic carriers thus ensure the persistence of *Leptospira* in the environment, to which accidental hosts are exposed. Among chronic carriers, the brown rat (*Rattus norvegicus*) is considered the main known reservoir for *Leptospira* infections [2, 15]. It plays a major epidemiological role in the transmission of leptospirosis as it is prevalent in urban areas and strongly linked to human activities. Indeed, the brown rat is a synanthropic species [16], requiring humans to maintain its population [17]. Additionally, it is also able to adapt rapidly and effectively to a range of environmental conditions. Consequently, it extensively populates urban environments, reaching considerable population densities in certain zones, particularly in areas of insalubrious and deteriorated infrastructures [18, 19], which offer favorable environmental conditions, including abundant food sources and readily accessible nesting areas. Because of its destructive behavior, its proliferation is the cause of numerous conflicts [17]. Currently, many large cities face the challenge of rat infestations which consequently increases the risk of leptospirosis. As highlighted by prevalence data reported in the literature [19–23], this risk varies significantly across different cities [19–23] and even within the same city [24]. Prevalence rates ranging from a few percent to almost 70% have been reported in the literature [19–23]. Nevertheless, available evidence suggests that age is a critical factor, with adult rats being more likely to be infected than subadults and juveniles [20, 22, 24]. The mode of intraspecific transmission may influence the importance of the “age” factor, depending on whether the rat is contaminated mainly via the environment or through contact with its congeners [25]. The difficulty of trapping brown rats in urban environments presents a significance challenge to the study of the dynamics of leptospirosis infections within the same brown rat population. However, this is a crucial question, and a more comprehensive understanding of the epidemiology of leptospirosis in brown rats could assist in curbing their role in the spread of the disease, by contributing to the implementation of appropriate surveillance program and possibly measures to prevent their contamination.

The aim of this study was (i) to describe the prevalence of *Leptospira* carriage in two different brown rat populations located in poor urban areas with high human population densities and under different climatic constraints, (ii) to identify the age-related influence on *Leptospira* carriage, and (iii) to identify the mode(s) of contamination within the rat population. To achieve this, an innovative and original trapping method was used to access the entire population, rather than a very limited and unrepresentative sample.

## 2. Materials and Methods

### 2.1. Ethics Status

This study was informed by pest control initiatives implemented by accredited social housing providers in response to numerous grievances from residents. The social landlords commissioned pest control operators (PCOs) to biologically manage the outbreaks without the use of rodenticides. This involved the deployment of ferrets to dislodge rats from their galleries and the utilization of trapping nets to apprehend them upon their emergence from their galleries.

After trapping, experimental procedures on the rats were performed according to an experimental protocol following international guidelines and with approval from the Ethics Committee of the Veterinary School of Lyon and French authorities (authorization n°#37713-2022042712262465). This study complied with the ethical standards of European regulations governing the care and use of animals in research (Directive 2010/63/EU), and it did not involve any endangered or protected species, or protected areas.

### 2.2. Study Area and Period of Trapping

The captures were carried out between February and June 2023 in social housing units with high human population densities, high levels of insalubrity, and high brown rat population densities, with numerous signs of presence and rats visible in vegetation areas during the day. Captures were made in all surrounding vegetated plots where rat galleries were visible, to capture all or almost all the rat population present in the vicinity of the housing. Two social housing sectors were included in the study.

**Figure 1:**
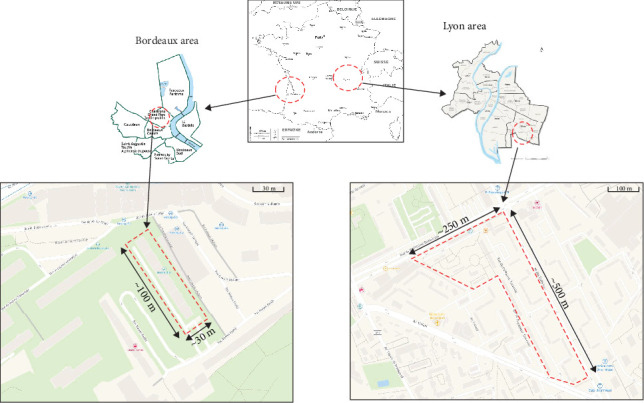
Trapping areas in Bordeaux and Lyon, France.

The largest area of social housings was located in the city of Lyon (more than 500,000 inhabitants), the capital of the Auvergne-Rhône-Alpes region (Figure 1, on the right). The city is located in the southeastern of France [26] and exhibits a semicontinental climate with Mediterranean influences, with more precipitation in summer than in winter. The social housing area was located in the 8th arrondissement, to the southeast of Lyon. The trapping area covered ~6 ha surrounding 10 buildings. The trapping was carried out over a period of 16 nonconsecutive days between February and June, with two to three trapping sessions per spot of vegetation in which galleries and/or signs of presence were detected. The last trapping confirmed that there were no more individuals in the spot. The second area of social housing was located in Bordeaux (more than 250,000 inhabitants), the capital of the Nouvelle-Aquitaine region (Figure 1, on the left). The city is located in the southwestern of France [26] and is characterized by an oceanic climate. The social housing area was located to the north of the Grand Parc district. The capture area was more limited in extent, covering around 0.3 ha around a single building. The trapping lasted a full week, with two to three trapping sessions per spot of vegetation. The last trapping confirmed that there were no more individuals in the spot.

### 2.3. Trapping Method

The rodents were captured by PCO commissioned by the social landlords. The PCO implemented an innovative biological trapping method that enabled us to capture all or almost all the rats present in our study areas. The rats were captured alive using specially trained ferrets (a total of 15 ferrets were used to cover all the areas). The first step consisted of detecting all the galleries present in the study areas. For trapping rats, after sealing off the area around the gallery(ies) with robust fine-meshed nets, the ferrets were released within the restricted perimeter. The ferrets infiltrated the tunnels, prompting the rats to abandon their burrows and resurface. The rats were then captured alive by the PCOs using landing nets. Once all the rats had been dislodged, the ferrets left the galleries. When all the ferrets had finished their hunting work, the galleries were dismantled by the PCOs using shovels and picks. This process was undertaken to ensure that all the rats had left the gallery and to retrieve any offspring remaining in the nests. The rats were then transported in individual cages to a laboratory in Lyon and Bordeaux to be anesthetized with isoflurane for blood taking and euthanized with CO_2_ for tissue sampling.

### 2.4. Collection of Individual Morphological Parameters

In the laboratory, the rats were promptly anesthetised with isoflurane and euthanized by cardiac puncture and cervical dislocation. In accordance with [27], a comprehensive set of data was collected for each animal including sex, weight, height, body length, and tail length. The height, body length, and tail length were all measured in centimeters with a precision of 0.5 cm. The weight was determined with an electronic scale with an accuracy of 0.5 g. The sex of the animal was established based on anogenital distance and/or observation of of the testicles and was subsequently verified at autopsy. All data, including the date and trapping location, were recorded into a database.

### 2.5. Sampling of Kidney Tissue and Possibly Urine and Embryos and Isolation of *Leptospira*

One kidney was excised, transferred to a sterile tube, and stored at −80°C until DNA extraction. To assess the potential for *Leptospira* excretion by the rats, urine when present was also collected via direct puncture in the bladder with a sterile needle and syringe. A portion of the urine was stored for subsequent DNA extraction.

Additionally, the second kidney of a series of rats was collected, crushed using a sterile single-use syringe and transferred, along with the second portion of the aseptically harvested urine, into Ellinghausen–McCullough–Johnson–Harris (EMJH) medium tubes for the isolation of *Leptospira*. After following dilution cascades (1/10, 1/100, and 1/1000), the culture tubes were incubated aerobically at 28°C–30°C for a period of 2 months, with weekly dark-field microscopic observation conducted to assess the potential presence and growth of *Leptospira* [28].

The embryos of all pregnant females were also meticulously individually collected to avoid contamination with maternal tissue. Their gestational stage was then determined in accordance with the procedure outlined by Butler [29]. When embryonic development permitted (embryo weight >3 g), the kidneys were isolated from the rest of the body. The embryos and kidneys from embryos were then placed in sterile tubes and stored at −80°C until DNA extraction for *Leptospira*-testing qPCR DNA extraction

Prior to extraction, sterile phosphate-buffered saline was used to rinse the kidneys and embryos to remove any environmental debris and contaminants [30]. Subsequently, a sterile single-use syringe was employed to grind them. The DNA was extracted from the kidney or embryo grindings as well as from the preserved urine portion, which had been preserved using the BioExtract SuperBall kit (Biosellal, Dardilly, France) in accordance with the manufacturer's guidelines. The efficacy of the DNA extraction process and the potential presence of inhibitors were evaluated through the quantification of the endogenous β-actin housekeeping gene using real-time PCR (qPCR). The DNA extracts were then stored at −20°C until used for *Leptospira* molecular analysis.

### 2.6. *Leptospira* Detection and Species Identification

The detection of pathogenic *Leptospira* was performed by qPCR targeting a partial region of the 16S rRNA gene using specific TaqMan primers and probe, as previously described [31]. The amplification reactions were performed using the AgPath-ID One-Step RT-PCR kit (Life Technologies, Courtaboeuf, Les Ulis, France) according to the manufacturer's instructions. All tests were conducted using the Mx3000P RT-PCR detection system (Agilent Technology, Courtaboeuf, Les Ulis, France). Each reaction was performed in a total volume of 25 µL containing 2× PCR buffer, 400 nM of each primer, 400 nM TaqMan probe, and 4 µL DNA. The amplification conditions were as follows: an initial denaturation phase at 95°C for 10 min, followed by 40 cycles of 15 s at 95°C for the amplification step, and 1 min at 60°C for the annealing phase.

*Leptospira*-positive samples were then subjected to further *L. interrogans* strain-specific qPCR, as previously described [32]. Amplification reactions were also carried out using the AgPath-ID One-Step RT-PCR kit (Life Technologies, Courtaboeuf, Les Ulis, France) according to the manufacturer's instructions and the Mx3000P RT-PCR detection system (Agilent Technology, Courtaboeuf, Les Ulis, France). For this PCR, a 25-µL reaction mixture was prepared containing 2× PCR buffer, 400 nM of each primer, 400 or 120 nM TaqMan probe, and 4 µL of DNA. The amplification program started with a denaturation step at 95°C for 10 min, followed by 40 cycles of 15 s at 95°C for the amplification phase and 1 min at 60°C or 63°C for the annealing phase. A positive control (DNA from the ENVN serovar Icterohaemorrhagiae strain of *L. interrogans*) and a negative control (4 µL of nuclease-free water) were included in each PCR protocol, and data analysis was performed using the detection system RT-PCR equipment according to the manufacturer's instructions.

### 2.7. Determination of Serogroup and Serovar of *Leptospira* for Positive Samples

For sample positive for *L. interrogans* carriage, the serogroup was identified through the amplification of the O antigen using primers that were specific to the serogroup Icterohaemorrhagiae [33]. Each reaction was performed using Platinum SuperFi II Green PCR Master Mix (Fisher Scientific, Illkirch, France), in a final volume of 20 µL containing 500 nM of each primer and 2 µL of DNA. Amplification was achieved by an initial denaturation at 98°C for 30 s followed by 35 cycles of denaturation at 98°C for 10 s, annealing at 60°C for 10 s, and polymerization at 72°C for 30 s, followed by a 5-min terminal elongation. A positive control (DNA from *L. interrogans* serovar Icterohaemorrhagiae ENVN) and a negative control (2 µL of nuclease-free water) were included for each analysis. The amplification products were subjected to electrophoresis on a 1% agarose gel. Fragments were visualized using a UV transilluminator, and their molecular weight was compared to a 100 bp ladder (Invitrogen) and to the positive control. PCR products were sequenced by Genoscreen (Lille, France) for serogroup confirmation.

The serovar was identified using the lic12008 gene-centered method. The presence of an insertion at the 5′ end of the lic12008 gene in serovar Icterohaemorrhagiae but not in serovar Copenhageni enabled the differentiation between both serovars [34]. The PCR amplification was carried out according to the same conditions than previously. The PCR products obtained, after verification by electrophoresis on 1% agarose gel, were sequenced by Genoscreen (Lille, France). The sequences were read using ChromasPro 2.6.6 software and analyzed using CLC sequence viewer 7.6.1 software.

### 2.8. Statistical Analysis

A statistical modeling approach was conducted to evaluate the potential of rat-related and urban criteria as predictors of leptospirosis infection in rats. A simple logistic regression (SLR) model based on a binomial distribution was employed to investigate the association between the prevalence of leptospirosis and the explanatory variables (weight, sex, months, and location). Subsequently, multivariate logistic regression models (generalized linear model [GLM]) based on a binomial distribution for prevalence were implemented, incorporating weight, sex, and location as variables (month was not included in the GLM as there were not enough different trapping period in Bordeaux). The best model was selected based on the Akaike information criterion (AIC) using the “glmulti” package [35]. Observations containing missing values were removed from the analysis. Variation inflation factors were calculated using the “car” package in R [36] to confirm the absence of significant collinearity (VIF < 5).

In the context of real-time PCR cycle threshold (Ct) analysis, a Kruskal–Wallis test was utilized, followed by a post hoc Dunn's test, to assess the impact of weight on this parameter. Furthermore, a multivariate linear regression model was constructed to identify the influence of city, sex, and weight. Normality of residuals and homoscedasticity were assessed graphically.

## 3. Results

### 3.1. Characteristics of Rat Populations

A total of 508 rats (*R. norvegicus*) were trapped and included in this study, comprising 407 rats trapped in the 6-ha trapping area in Lyon and 101 rats trapped in the 0.3-ha trapping area in Bordeaux. The rats were confirmed to belong to the *R. norvegicus* species through morphological measurements and cytochrome b sequencing. The “Lyon” sampling group consisted of 180 female rats, 180 male rats, and 47 juvenile rats whose sex was not determined. The median weight of mature adult rats (i.e., >200 g) was 362.8 g (CI_95%_: 349.4–381.9) with median head/body and tail lengths of 22.5 cm (CI_95%_: 22.0–22.9) and 19.0 cm (CI_95%_: 19.0–19.5), respectively. The “Bordeaux” sampling group consisted of 48 female rats and 53 male rats. The median weight of mature adult rats (i.e., >200 g) was 347.1 g (CI_95%_: 288.9–394.6) with median head/body and tail lengths of 22.0 cm (CI_95%_: 21.0–23.0) and 20.5 cm (CI_95%_: 19.0–21.0), respectively. Rats of all ages (from newborns to very old adults) were present at both sites and were divided by weight category (Table 1). The proportions of animals between weight categories were not statistically significantly different between spots of trapping (*X*^2^ = 11.74, df = 6, *p*=0.0681).

### 3.2. Overall Prevalence of *Leptospira* Infection in Rat Populations in the Study Areas

Based on qPCR targeting a partial region of the 16S rRNA gene performed from kidney DNA, 210 of the 407 rats trapped in the study area of Lyon were positive for *Leptospira* DNA, resulting in an overall prevalence of 51.8% (CI_95%_: 47.4–56.3). In the study area of Bordeaux, 29 of the 101 rats were positive for *Leptospira* DNA, resulting in an overall prevalence of 28.7% (CI_95%_: 20.8–38.2).

For rats captured in the Lyon study area, *Leptospira* were successfully cultured from the tissues (kidneys and urine) of 22 rats (13 females and 9 males) out of 157 (14% [CI95%: 9.4–20.3]) for which culture was performed. Cultures that were considered negative were either those in which no *Leptospira* was detected after a 10-week incubation period at 30°C or cultures in which contaminants were rapidly detected following inoculation, thereby preventing the detection of any *Leptospira*. Among the positive cultures, two were from rats with a weight higher than 500 g (*n* = 9), six from rats with a weight between 400 and 500 g (*n* = 44), 10 from rats with a weight between 300 and 400 g (*n* = 54), and four from rats with a weight between 200 and 300 g (*n* = 24). The remaining rats with a weight lower than 200 g (*n* = 26) yielded no positive positive results. Among the rats with a positive culture result, two were not detected as positive by qPCR. For other, Ct values obtained by qPCR were comprised between 17.4 and 39.5.

For rats captured in the Bordeaux study area, *Leptospira* were successfully cultured from the tissues (kidneys and urine) of a single female rat weighing over 500 g. This rat was also found positive by qPCR. This low success rate was attributed to a high rate of contamination by *Proteus* and *E. coli*.

Considering the positive results obtained by qPCR and culture, the overall prevalence in Bordeaux remained unchanged from that calculated on the basis of qPCR results, that is, 28.7% (CI_95%_: 20.8–38.2). In Lyon, the overall prevalence increased slightly, reaching 52.1% (CI_95%_: 47.2–56.9).

### 3.3. Molecular Typing of *Leptospira* Strain in *R. norvegicus* in Lyon and Bordeaux Study Areas

*A L. interrogans*-specific qPCR was applied for the analysis of all 239 positive samples. All rats that tested positive for *Leptospira* were found to be positive for *L. interrogans*, regardless of their location.

O-antigen typing was applied to DNA obtained from the kidneys (positive samples) and successful cultures with positive controls confirming the robustness of the results. The O typing revealed the presence of the serogroup Icterohaemorrhagiae in all samples analyzed, in both the Lyon and Bordeaux study areas. The diagnostic marker lic12008 enabled the identification of the serovar of the strain in positive rats (positive samples + successful cultures) from both cities, which was determined to be serovar Icterohaemorrhagiae.

### 3.4. Infection Status of Rats According to Weight Category, Sex, Period of Trapping, and City

**Figure 2:**
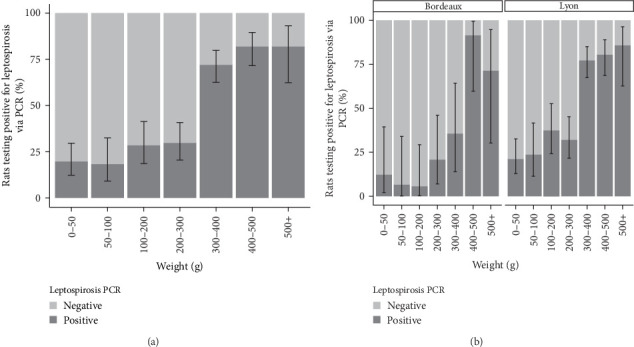
Evolution of the prevalences of *Leptospira* infection in trapped rats according to the weight categories. In (A) globalizing all the rats included in the study and (B) considering only the rats included in the study area of Lyon and in the study area of Bordeaux.

The analysis of infection status was conducted on the basis of weight categories. The results are presented in Figure 2. In the Lyon study area, the *Leptospira* prevalence was 28.4% (CI_95%_: 22.8–34.6) among individuals with a weight below 300 g. The prevalence exceeded 80% for individuals with a weight above 300 g. In Bordeaux, the prevalence of *Leptospira* was below 35% for all weight categories except those weighing over 400 g, where carriage exceeded 80%.

**Figure 3:**
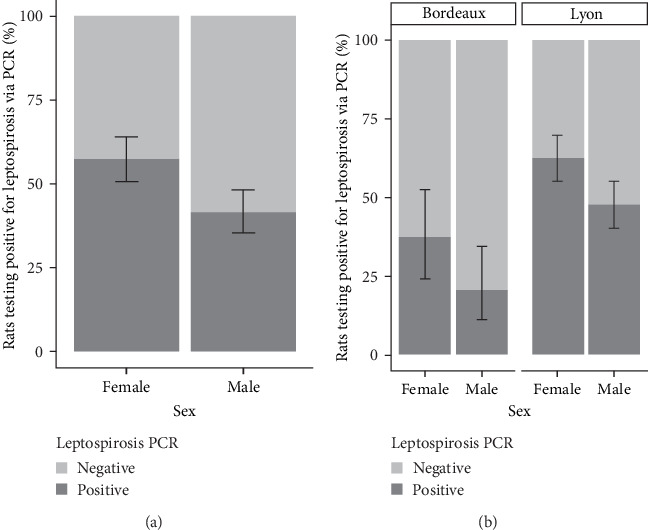
Prevalences of *Leptospira* infection in trapped rats according to the sex. In (A) globalizing all the rats included in the study and (B) considering only the rats included in the study area of Lyon and in the study area of Bordeaux.

The analysis of infection status was also conducted according to sex. The results are presented in Figure 3. A greater proportion of females were found to be positive for *Leptospira* in both the study areas of Lyon and Bordeaux.

To facilitate a statistical analysis of the results, the weight categories were grouped based on the trend of prevalence within each category. Consequently, the redefinition has resulted in the creation of four weight categories: 0–200 g, 200–300 g, 300–400 g, and over 400 g. The SLR model revealed that gender was not a factor influencing *Leptospira* positivity, regardless of city. On the other hand, the probability of being positive increased with increasing weight (Table 2A).

The best model in terms of AIC was the one considering location, sex, and weight without interaction (AIC = 497.1). The second model considered the same variables but included an interaction between weight and location (AIC = 497.4). However, this interaction was not statistically significant (*p*=0.128), and thus the first model was retained. In this GLM, there was an increase in *Leptospira* positivity with higher weight. This positivity was also different between study areas (Table 2B). However, there was no discernible difference in the correlation between sex and *Leptospira* positivity.

### 3.5. Analysis of the *Ct* Values Obtained by PCRq Targeting a Partial Region of the 16S rRNA Gene in Positive Rats According to Their Weight Categories

**Figure 4:**
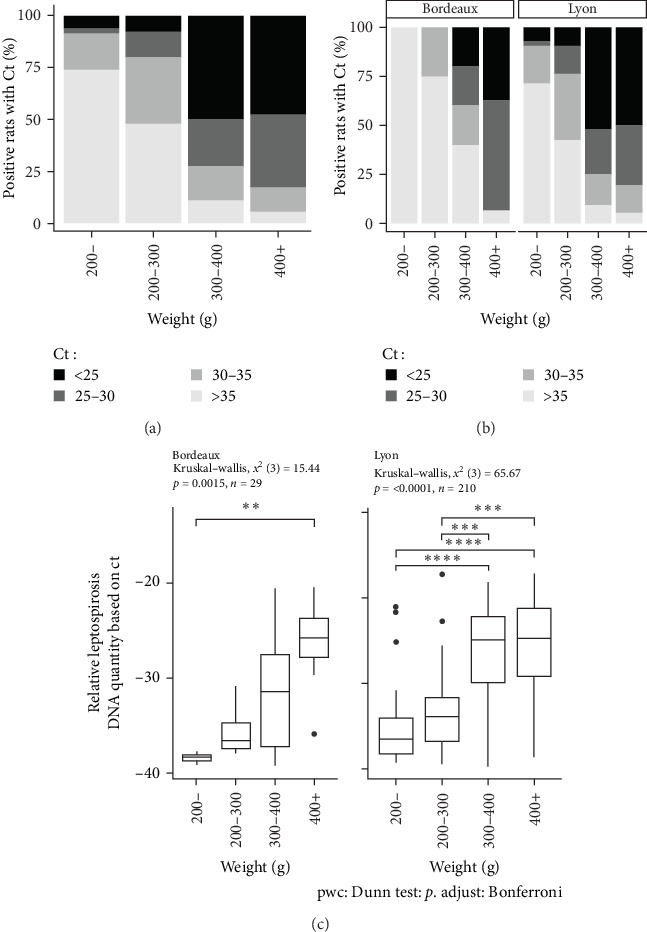
Distribution of *Leptospira*-positive rats according to their Ct value and median Ct value by weight category. (A) Distribution of rats according to their Ct, including all the positive rats of the study. (B) Distribution of rats according to their Ct, including only the positive rats detected in the study area of Lyon or in the study area of Bordeaux. (C) Relative leptospirosis DNA quantity based on median Ct value by weight category. Statistical analysis of Ct values between weight categories was done using Kruskal–Wallis test, followed by pairwise comparison using Dunn's test with a Bonferroni adjustment (*p*-value <0.05, difference statistically significant). *⁣*^*∗*^*p*-value <0.05, *⁣*^*∗∗*^*p*-value <0.01, *⁣*^*∗∗∗*^*p*-value <0.001, and *⁣*^*∗∗∗∗*^*p*-value <0.0001.

The Ct is defined as the number of cycles required for the fluorescence signal to exceed the background level. The lower the Ct is, the higher the bacterial load is. Positive animals were grouped by Ct category (i.e., animals with a Ct value <25, between 25 and 30, and between 30 and 35 and >35) and by weight category (animals weighing less than 200 g were grouped together, due to the lower number of positive individuals in these categories). The percentages of positive animals in each category are shown in Figure 4. Based on the qPCR results, and after considering the new weight categories, it was evident that the PCR Ct value characterizing bacterial load depended on weight rather than city. Most of the *Leptospira*-positive animals in the higher weight categories had a PCR Ct value below 30 in both the study areas of Lyon and Bordeaux (Figure 4B). On the contrary, in rat categories weighing less than 200 g, Ct values were predominantly higher than 35.

In the study area of Bordeaux, a significant difference in Ct was observed according to the animal weight (between animals weighing more than 400 g and those weighing less than 200 g (Kruskal–Wallis, *X*^2^ (3) = 15.44, *p*=0, 0015; post hoc Dunn test <200 g vs. >400 g, *p*  < 0, 01) (Figure 4C). In the study area of Lyon where the number of trapped animals was higher, significant differences were observed between animals weighing more than 300 g and those weighing less than 300 g (Kruskal–Wallis, *X*^2^ (3) = 65.67, *p*  < 0, 0001; post hoc Dunn test <200 g vs. 300–400 g or >400 g: *p*  < 0, 001 and 200–300 g vs. 300–400 g or >400 g *p*  < 0, 0001) (Figure 4C).

In the multivariate linear regression model, the weight factor had a significant effect on the Ct PCR value compared to sex (Table 3).

### 3.6. *Leptospira* Infection Status of Rat Embryos

In the Lyon study area, 32 out of 180 females (17.8%) were pregnant, including 14 weighing over 300 g, 6 over 400 g, and 9 over 500 g. Of the 32 females, 28 were positive for *Leptospira* carriage. Litters from 13 positive females were selected on the basis of maternal weight and Ct value of *Leptospira*-testing qPCR performed on females and embryo size. The total number of embryos collected from these females was 98, with an average number of embryos per female of 7.5 ± 2.6 (Table 4). The smallest embryos were at least 1 cm in size, indicating that all females were in the late stages of gestation. Unlike the dams, all the embryos or embryonic kidneys were negative for PCR-tested *Leptospira* carriage.

## 4. Discussion

This study provides valuable insights into the persistence and transmission of *Leptospira* at the scale of urban rat colonies/populations. Its focus on this specific objective contributes to its novelty. Indeed, most research on *Leptospira* carriage in urban brown rats has aimed to provide a broad-scale view of this risk within a city. To achieve this broad perspective, these studies have typically relied on a consistent field approach, with punctual sampling of rats by mechanical trapping at multiple distinct sites spread over large areas, resulting in limited sample sizes per site [20–24, 37, 38]. The focus on the colony/population scale necessitated a different methodology that could capture an entire colony/population. Hence, our study focused on two distinct urban sites, with relatively limited areas, ~6 ha in Lyon and ~ 0.3 ha in Bordeaux, where brown rats were proliferating. The colony-scale perspective required the capture of the whole or almost the whole population. The trapping technique employed was completely original and innovative but not applicable in all ecosystems, such as sewers, indoors, in more built-up areas. The method involved the use of ferrets to penetrate all burrows and drive the rats to the surface, where they were then all manually captured with nets. The systematic destruction of the burrows using gardening tools subsequently allowed for the recovery of any remaining, newborn and younger rats from the colony. All burrows within the study areas were identified and surveyed in this manner.

Using this capture method, all or almost all the animals present in the study area could be captured and included in the study. Four hundred seven rats were captured at the Lyon site, that is, to 68 rats/ha, and 101 rats at the Bordeaux site, that is, 336 rats/ha. *Leptospira* carriage was analyzed by two parallel methods: bacteriological culture of one kidney and/or urine and PCR detection from the other kidney [39]. While bacteriological culture provides a definite, early diagnosis [40], that is, compatible with advanced characterization of the bacteria [41], demonstrating the animal's involvement in environmental contamination through the excretion of viable *Leptospira*, PCR diagnosis remains essential for a relevant evaluation of carriage. Indeed, culture is a less sensitive method for detecting *Leptospira*, with generally low isolation frequency due to the slow growth of *Leptospira* and the presence of numerous other contaminants, particularly in rats. For this reason, in our study, an animal was considered positive if the PCR was positive, even if its culture was negative. However, since PCR can also be affected by the state of the animals' conservation, freeze–thaw cycles, and the presence of inhibitors, animals were captured alive and autopsied immediately after euthanasia to minimize the false negative rate [42].

This study thus advances our understanding of the dynamics of pathogenic *Leptospira* of the interrogans species in urban brown rats and progresses our knowledge of the transmission pathways of *Leptospira* within a rat population through the analysis of variations in leptospirosis carriage prevalence according to age. The methodology adopted allowed us to explore this by capturing all animals regardless of their age, whereas in traditional studies, capture by mechanical trapping obviously excludes young animals that do not stray far from the nest and probably the oldest, neophobic animals that are reluctant to enter a confined wire space. Because of the challenge in accurately determining the animals' ages, we classified them based by weight (<50 g, 50–100 g, 100–200 g, 200–300 g, 300–400 g, 400–500 g, and >500 g) although aware of the biases inherent in the choice, as weight and age are not linearly related. Some authors have proposed an estimate of rat age based on weight [43] using the von Bertalanffy equation for growth [44] and have generated a standardized curve for wild rats using weight and age data obtained from captive colonies of wild rats [45]. Unfortunately, this equation is not applicable to young rats less than 1 month old, for which the calculated weight would not be consistent with the real weight (a 23-day-old rat would weigh 0 g).

The proportions of captured animals were relatively comparable across the different weight categories. For our interpretation, we will consider that rats under 50 g are confined to the nest. Once weaned, they leave the nest and begin to move freely as subadults, finally reaching sexual maturity between 150 and 200 g. Whether in Lyon or Bordeaux, our results show an increase in prevalence with the weight of the animals and consequently with the age, as already suggested by many studies [20, 22, 25, 46]. Surprisingly, in both Lyon and Bordeaux, our study reveals a sudden change in prevalence at 300/400 g, jumping from 10 to 20% to over 75%. This sudden shift demonstrates the major role of horizontal and environmental transmission in the dynamics of leptospirosis infections. At 300–400 g, rats exhibit exploratory behavior toward their environment and aggressive behavior toward their conspecifics during food foraging, defense, and acquisition of new territories or while seeking sexual partners [47]. Multiple bites may follow [48], facilitating the transmission of *Leptospira* via the environment or the conspecific [46]. Besides the high prevalence rate observed in rats weighing over 300 g, these rats also had the highest bacterial loads in the kidney, as shown by Ct values [49]. This observation indicates a sustained carriage but, more importantly, establishes the absence of pathogenic consequences of *Leptospira* infection in rats [40]. It would be interesting to establish this same relationship from urine, to assess urinary excretion of *Leptospira* by age and to evaluate the role of different animal categories in environmental contamination. Previous studies suggest that urinary leptospiral loads also increased with the age of rats [50].

Although the prevalence of carriage in young rats is significantly lower, in the order of 10% to 20%, our results demonstrate that they can also be infected. In utero transmission has been suggested [51] and confirmed later by isolating *Leptospira* Icterohaemorrhagiae from fetuses [52]. However, our findings do not seem to support in utero transmission, as all embryos collected from positive pregnant females were PCR negative. Nevertheless, it is important to note that our embryos were stored at −80°C for several weeks. Long-term storage can lead to decreased DNA concentrations, which may have reduced the chances of detecting *Leptospira* DNA in our embryos, as observed in other studies [24, 42]. Experimental studies will be necessary to definitively establish whether in utero transmission occurs or not. Breast milk transmission has also been suggested [53] by the detection of the presence of *Leptospira* in milk and mammary tissue. However, this mode of transmission does not appear to have a significant epidemiological impact according to our results. Additionally, it is possible that the young rats in which bacteria were found had lost their immunity, as maternal antibody transfer seems to delay infection [54]. Besides transmission through milk, infant contamination can occur through the urine or saliva of the infected mother, during urogenital grooming and cleaning of her pups. Apart from these vertical or pseudovertical contaminations, transmission of bacteria among young individuals can occur in various ways, including through social play. During social play, young rats learn and imitate adult behaviors [55], increasing their exposure to contaminated environments. Nevertheless, our results demonstrate that infection of young rats by vertical or horizontal transmission seems to be negligible.

In addition to advances in understanding the transmission pathways and the dynamics of *Leptospira* infection in brown rats, our study has demonstrated in Bordeaux and confirmed in Lyon the maintenance and circulation of *L. interrogans* among brown rats. It also facilitated the precise characterization of the circulating strain. A single strain was identified in both populations studied: *L. interrogans* belonging to Icterohaemorrhagiae serogroup and Icterohaemorrhagiae serovar. This finding confirms the reservoir role played by rats and supports the idea that a specific strain of *Leptospira* is associated with a specific rat colony [24]. The study also allowed for the measurement of *Leptospira* carriage prevalences that can be qualified as “real,” as opposed to the “relative” prevalences reported in most studies, which rely on large-scale sampling influenced by the positioning of traps and the characteristics of the individuals captured. Prevalence rates reached 52% at the Lyon site and 22% at the Bordeaux site. The prevalence measured at the Lyon site seems high compared to other French urban areas. In Paris, a recent study indicates a relative prevalence of 15% [56], while another, older study in the Paris suburbs reports a prevalence of 21% [37]. However, when comparing the prevalence measured at the Lyon site with those reported in a previous study, it is within the upper range of previously reported values, varying from 0% to 65% depending on location [24]. This previous study suggested a correlation between leptospirosis prevalence, population density, and income level. Our Lyon site, characterized by a high population density and a low economic income, confirms this hypothesis. The lower prevalence determined at the Bordeaux site, comparable to Parisian results, raises questions despite demographic and economic conditions similar to those of the Lyon site. On the Lyon site, the rats' increased exposure to a contaminated environment, notably through access to sewers, could explain the high prevalence. A study in the sewers of Copenhagen indeed reveals higher prevalences in sewer rats [22]. Future research should explore this hypothesis. Nonetheless, the difference in prevalence between the Lyon and Bordeaux sites must be nuanced. Indeed, prevalences among older animals were identical, around 75%. The overall prevalence difference between the Lyon and Bordeaux sites could easily be explained by the difference in population structure, with a proportionally higher number of rats over 300 g in Lyon (45%) than in Bordeaux (33%). This underscores the importance of interpreting and cautiously comparing relative prevalences that entirely depend on the structure of the sampling. For studying the leptospirosis risk associated with urban rats at a site, would not it be important to only consider animals over 300 g, which more accurately reflect environmental contamination? This assessment should thus consider (i) the human population density and its proximity to rats, (ii) the rat population density, (iii) the identification of the circulating strain, and (iv) the prevalence of carriage in rats over 300 g or the overall carriage prevalence confronted with the structure of the captured rat population. The detection of *L. interrogans* at the Lyon and Bordeaux sites, while this strain is the main strain involved in severe cases of leptospirosis in humans, underlines the infection risk to which urban populations might be exposed. This consideration takes on a particularly critical dimension considering the observed carriage prevalences and the fact that the capture sites, both in Lyon and Bordeaux, were in areas with high population density and characterized by environmental conditions favorable to rat proliferation (food availability, presence of water, and vegetated ground allowing the formation of galleries and nesting, without real predators). Consequently, this results in an increased probability of human exposure to this pathogenic strain in these urban areas.

## Figures and Tables

**Table 1:** Characteristics of rat populations according to sex and weight class.

Population of rats		6-ha trapping area in Lyon *n* = 407	0.3-ha trapping area in Bordeaux *n* = 101
Sex ratio		Males: 44.2% (*n* = 180) Females: 44.2% (*n* = 180) Not identified: 11.6% (*n* = 47)	Males: 52.5% (*n* = 53) Females: 47.5% (*n* = 48)

Weight class	0–50 g	18.4% (*n* = 75)	15.8% (*n* = 16)
	50–100 g	8.4% (*n* = 34)	14.9% (*n* = 15)
	100–200 g	11.8% (*n* = 48)	17.8% (*n* = 18)
	200–300 g	16.0% (*n* = 65)	18.8% (*n* = 19)
	300–400 g	23.8% (*n* = 97)	13.9% (*n* = 14)
	400–500 g	16.5% (*n* = 67)	11.9% (*n* = 12)
	>500 g	5.2% (*n* = 21)	6.9% (*n* = 7)

**Table 2:** Odds ratio (OR) using (A) the simple regression logistic (SRL) model for testing positive for *Leptospira interrogans* to determine factors affecting *Leptospira* prevalence by study areas, (B) using the generalized linear model (GLM) for testing positive for *Leptospira interrogans* among *Rattus norvegicus* included in the study, and incorporating the study area, the sex, and the weight category.

(A) Odds ratio using the SRL model								
		Lyon study area				Bordeaux study area		
Covariates		OR	CI_95%_	*p*		OR	CI_95%_	*p*
Sex	Female	Ref	—	—		Ref	—	—
	Male	0.48	0.32–0.70	<0.001		0.44	0.17–1.04	0.066

Weight	<200 g	Ref	—	—		Ref	—	—
	200–300 g	1.39	0.77–2.49	0.268		3.00	0.64–14.2	0.152
	300–400 g	11	6.51–19.3	<0.001		6.25	1.4–30	0.016
	>400 g	13.8	7.78–25.5	<0.001		60	13.9–362	<0.001

Month of trapping	April	Ref	—	—		—	—	—
	February	0.57	0.35–0.91	0.020		—	—	—
	June	0.41	0.06–2.16	0.313		—	—	—
	March	1.0	0.69–1.53	0.902		—	—	—

**(B) Odds ratio using the GLM model**								
**Covariates**		**OR**		**CI_95%_**		***p***		

Study area	Bordeaux area	Ref		—		—		
	Lyon area	2.76		1.58–4.92		<0.001		

Sex	Female	Ref		—		—		
	Male	0.66		0.42–1.03		0.06		

Weight	<200 g	Ref		—		—		
	200–300 g	1.32		0.57–1.88		0.38		
	300–400 g	7.67		4.47–13.79		<0.001		
	>400 g	14.93		8.06–28.85		<0.001		

*Note*: *p*-value <0.05: statistically significant compared to corresponding reference (ref). Odd ratios greater than 1 indicate an increased risk of *Leptospira interrogans* carriage for these factor levels compared to the reference level according to the model.

**Table 3:** Estimates using the multivariate linear regression model for relative *Leptospira* DNA quantity based on Ct values obtained with the *Leptospira*-testing qPCR targeting a partial region of the 16S rRNA gene incorporating the study area, the sex, and the weight category for *R. norvegicus* with Ct <40.

Covariates		Estimate	CI_95%_	*p*
Study area	Bordeaux area	Ref	—	—
	Lyon area	1.99	−0.21–4.19	0.08

Sex	Female	Ref	—	—
	Male	−0.19	−1.67–1.30	0.81

Weight	<200 g	Ref	—	—
	200–300 g	1.37	−1.51–4.26	0.35
	300–400 g	8.06	5.82–10.30	<0.001
	>400 g	9.03	6.82–11.23	<0.001

*Note:* Multivariate linear regression model; *p*-value <0.05, statistically significant compared to the reference. Estimate greater than 0 indicates an increased risk of leptospirosis DNA quantity for these factor levels compared to the reference level according to the model.

**Table 4:** Characteristics of pregnant females and their litter used for *Leptospira*-testing qPCR.

Pregnant female identity	Weight category	Ct *Leptospira*-testing qPCR for female	*n* embryos	Weight (g) (mean ± SD)
Female 1	300–400	26.10	6	0.95 ± 0.05
Female 2	300–400	25.93	8	0.95 ± 0.08
Female 3	300–400	26.89	9	4.86 ± 0.11
Female 4	300–400	37.11	9	1.84 ± 0.05
Female 5	300–400	20.47	5	2.12 ± 0.08
Female 6	300–400	23.99	9	1.49 ± 0.11
Female 7	300–400	25.01	1	6.00 ± 0.00
Female 8	400–500	17.77	9	1.76 ± 0.09
Female 9	400–500	30.17	9	1.71 ± 0.10
Female 10	400–500	29.11	8	2.84 ± 0.07
Female 11	400–500	38.75	5	1.50 ± 0.00
Female 12	500–600	26.17	10	3.28 ± 0.17
Female 13	600–700	28.30	10	3.16 ± 0.13

## Data Availability

The data that support the findings of this study are available from the corresponding author, Virginie Lattard, upon reasonable request.
